# Human CD4^+^ T Helper Cell Responses after Tick-Borne Encephalitis Vaccination and Infection

**DOI:** 10.1371/journal.pone.0140545

**Published:** 2015-10-14

**Authors:** Judith H. Aberle, Julia Schwaiger, Stephan W. Aberle, Karin Stiasny, Ondrej Scheinost, Michael Kundi, Vaclav Chmelik, Franz X. Heinz

**Affiliations:** 1 Department of Virology, Medical University of Vienna, Vienna, Austria; 2 Laboratory of Molecular Genetics, Hospital České Budĕjovice, České Budĕjovice, Czech Republic; 3 Centre for Public Health, Medical University of Vienna, Vienna, Austria; 4 Department of Infectious Diseases, Hospital České Budĕjovice, České Budĕjovice, Czech Republic; Icahn School of Medicine at Mount Sinai, UNITED STATES

## Abstract

Tick-borne encephalitis virus (TBEV) is a human-pathogenic flavivirus that is endemic in large parts of Europe and Asia and causes severe neuroinvasive illness. A formalin-inactivated vaccine induces strong neutralizing antibody responses and confers protection from TBE disease. CD4^+^ T cell responses are essential for neutralizing antibody production, but data on the functionalities of TBEV-specific CD4^+^ T cells in response to vaccination or infection are lacking. This study provides a comprehensive analysis of the cytokine patterns of CD4^+^ T cell responses in 20 humans after TBE vaccination in comparison to those in 18 patients with TBEV infection. Specifically, Th1-specific cytokines (IFN-γ, IL-2, TNF-α), CD40 ligand and the Th1 lineage-specifying transcription factor Tbet were determined upon stimulation with peptides covering the TBEV structural proteins contained in the vaccine (C-capsid, prM/M-membrane and E-envelope). We show that TBEV-specific CD4^+^ T cell responses are polyfunctional, but the cytokine patterns after vaccination differed from those after infection. TBE vaccine responses were characterized by lower IFN-γ responses and high proportions of TNF-α^+^IL-2^+^ cells. In vaccine-induced responses—consistent with the reduced IFN-γ expression patterns—less than 50% of TBEV peptides were detected by IFN-γ^+^ cells as compared to 96% detected by IL-2^+^ cells, indicating that the single use of IFN-γ as a read-out strongly underestimates the magnitude and breadth of such responses. The results provide important insights into the functionalities of CD4^+^ T cells that coordinate vaccine responses and have direct implications for future studies that address epitope specificity and breadth of these responses.

## Introduction

TBEV is a human-pathogenic flavivirus that causes a significant public health problem with more than 10,000 annual cases of meningitis, encephalitis and/or radiculitis [1]. Inactivated, whole-virus vaccines are available and proved to be protective against TBE disease [2]. Long-term protection is thought to be primarily mediated by neutralizing antibodies [3]. CD4^+^ T cells are essential for helping antibody production by B cells, but detailed data on the functionalities of TBEV-specific CD4^+^ T cells in response to infection or vaccination are lacking.

As a member of the genus *flavivirus* in the family of *Flaviviridae*, TBEV is a close relative of other important arthropod-borne flaviviruses that cause yellow fever (YF), dengue fever (DF), Japanese encephalitis (JE), and West Nile fever (WNF) [4]. Flaviviruses are positive-strand RNA viruses that encode three structural proteins, the capsid (C), precursor of membrane (prM) and envelope (E) [5]. E is the primary target for neutralizing antibodies believed to be responsible for long-term immunity after vaccination and natural infection [6, 7]. CD4^+^ T cells recognizing epitopes on B cells can provide help for E-specific antibody production by direct B-T cell interaction. Since B cells can internalize whole virus particles [8–11], peptides derived not only from E but also from C and M can function as T-helper (Th) epitopes for B cells secreting E-specific neutralizing antibodies.

Human CD4^+^ T cell responses to flavivirus infection display a Th1 cytokine pattern, characterized by expression of interferon gamma (IFN-γ), interleukin-2 (IL-2) and tumor necrosis factor alpha (TNF-α) [12–16]. TBE vaccination also induces robust Th1 responses [17], which, after booster vaccination and with respect to IL-2 [18], were significantly higher than that after natural infection. However, it is unclear whether these responses differ in their cytokine composition. While all Th1 lineage cells express the Tbox transcription factor Tbet [19], the expression of cytokines during stimulation of Th1 cell populations is heterogenous and can impact the effector function of individual cells [20] as well as their capacity to develop into memory cells [21]. Th1 cells secreting all three cytokines (IFN-γ, TNF-α and IL-2) produce considerably more IFN-γ than single IFN-γ-secreting cells, and previous studies demonstrated that multi-cytokine producing cells are more effective at controlling a virus infection than single-cytokine producers [22–28]. Th1 subtypes that produce IL-2 have a greater capacity than single IFN-γ producers to proliferate [29] and to develop into long-term memory cells that can give rise to secondary effector cells following antigen re-challenge [30–34]. Thus, the extent to which individual Th1 lineage cells contribute in an overall response may be particularly important for maintaining an appropriate balance between cells exerting immediate effector functions and those providing a long-term memory pool [35].

So far, the distribution of Th1 lineage subtypes has not been addressed in the context of flavivirus vaccines. In the present study, we have therefore characterized the functional properties of Th1 responses in the TBEV system which allows a direct comparison of human Th cells generated in response to an inactivated vaccine and those generated during natural infection. We have used pools of peptides covering the entire sequence of each of the structural proteins C, prM/M and E to test CD4^+^ T cell responses in 20 persons after TBE vaccination and 18 patients with TBEV infection. We show that TBEV-specific CD4^+^ T cells induced after vaccination and infection are polyfunctional Th1 cells, but the cytokine patterns after vaccination are different from those after natural infection. Vaccination-specific patterns are characterized by i) a lower proportion of triple-positive cells as well as a reduced capacity of these cells to produce IFN-γ, and ii) a larger proportion of TNF-α^+^IL-2^+^IFN-γ^-^ cells that expressed reduced Th1 lineage-specifying transcription factor Tbet. We demonstrate that the lower IFN-γ expression pattern in TBE vaccine responses resulted in a 50% reduction of the number of TBEV E peptides detected with IFN-γ^+^ as compared to IL-2^+^ cells, indicating that a significant number of epitopes would have been missed using IFN-γ measurements only. The results, therefore, have important implications both for the identification of the mechanisms that contribute to protective immunity and the analysis of epitope specificity and breadth of these responses.

## Material and Methods

### Ethics Statement

The studies were performed in compliance with the provisions of the Declaration of Helsinki and its amendments and were approved by the ethics committees of the Medical University of Vienna, Austria (approval no. 590/2007) and the Hospital Ceske Budejovice, Czech Republic (approval no. 8/2008), respectively. Written informed consent for participation was obtained from all study participants.

### Human peripheral blood samples

Analysis of CD4^+^ T cell responses was performed with human peripheral blood mononuclear cells (PBMC) collected in citrate tubes (S-Monovette^®^ 10ml, Citrate 3.2%, Sarstedt, Germany). Blood samples were derived from 20 healthy Caucasians (mean age 49.1 years; range 21–74 years; 7M, 13F) previously vaccinated against TBE with a formalin-inactivated TBE virus vaccine (FSME-Immun^®^ 0.5 ml, Baxter) who were recruited at the Department of Virology, Vienna, Austria, as well as from a subgroup of Caucasian TBE patients (n = 18; mean age 49.9 years; range 23–81 years; 11M, 7F) hospitalized with clinical symptoms of acute TBE at the hospital Ceske Budejovice, Czech Republic, who had been enrolled in a T cell epitope mapping study conducted at the Department of Virology, Medical University of Vienna [18]. PBMC samples from patients hospitalized with acute TBE were taken 2.3 weeks after onset of first symptoms (range 1–4 weeks), and additional 5 samples were obtained from individuals with past TBEV infection, collected 1.5 months to 23 years after TBE. Samples from TBE vaccinees had been collected 1 to 7 weeks after booster vaccination (S1 Table).

For a comparison of primary and booster TBE vaccine responses, Boolean analysis was performed with flow cytometry data derived from samples obtained from 29 subjects who had been enrolled in a primary TBE vaccination study [17]. The mean age of the primary vaccinated subjects was 49.8 years; range 20–80 years, 16 were male and 13 female. PBMC samples were collected 1 week after 2-dose primary immunization (median, range 1–4 weeks).

### Isolation of peripheral blood lymphocytes

Mononuclear cells were isolated from peripheral blood using Ficoll-Paque^®^ PLUS, as recommended by the manufacturer (GE Healthcare), and stored in liquid nitrogen until tested. Plasma was obtained by centrifugation of the sodium-citrate-treated blood at 1600xg for 10 min and stored at -20°C.

### Peptides for identification of TBEV-specific CD4^+^ T-cell responses

A total of 188 15-mer peptides overlapping by 11 amino acids which cover the entire amino acid sequences of the C, prM/M and E proteins from TBE virus Neudörfl strain (NCBI 27596775, 27596776, 27596778) were purchased from jpt (Berlin, Germany). The peptides were grouped into 3 Maxipools, containing all peptides that covered each of either protein (E: 122, prM/M: 40 and C: 26 single peptides). In addition responses were analysed after stimulation with 11 TBEV E minipools that contained 11–12 single peptides. Peptides were used at a final concentration of 8 μg/ ml.

### Intracellular cytokine staining

To determine TBEV-specific Th1 subtype responses, PBMC were thawed and incubated overnight at 37°C in a 5% CO_2_ atmosphere. Aliquots of 500 μl containing 2 x 10^6^ PBMC were placed in 15-ml Falcon tubes (BD) and stimulated in the presence of 1 μg/ml anti-CD28/49d antibody (BD Pharmingen, USA) with either a TBEV peptide pool at 8 μg/ml or without peptides for 6 h at 37°C, 5% CO_2_, in the presence of 2 μM monensin (Sigma) and 40 μl PE-labelled anti-CD154 (BD Pharmingen) according to a protocol described previously [36]. After stimulation, cells were washed and stained using a LIVE/DEAD^®^ Cell Viability Assay Kit (Invitrogen) according to the manufacturer´s instructions to allow the exclusion of dead cells. After surface staining, cells were fixed and permeabilized using a Caltag Laboratories Fix&Perm^®^ cell permeabilization kit (Invitrogen) as recommended by the manufacturer. Following permeabilization, cells were stained for intracellular cytokines, as recommended by the manufacturer (S2 Table). Intracellular staining with the anti-Tbet-PE (e-Bioscience) was performed using the FoxP3 staining buffer set (eBioscience), as recommended by the manufacturer. The number of CD4^+^ T lymphocyte-gated events ranged between 80,000 and 500,000 in all experiments. Cells were analysed on a FACS Canto^TM^ II (BD, USA) cytometer. Data were further analysed using BD Diva software version 6.1.2 or FlowJo software version 7.2.5 (Tree Star, USA). Boolean gating function of FlowJo was used to assess each possible cytokine combination. The gates for detection of IL-2, TNF-α, IFN-γ in peptide-stimulated cell samples were set in the samples with costimulation only; as reported by others [37], single TNF-α subsets show high background staining, and were therefore not considered positive in Boolean analysis. Responses obtained by Boolean analysis were considered positive if they were ≥ twofold above the mean of samples with no antigen stimulation and at least 100 events/10^6^ CD4^+^ T cells after background subtraction.

### ELISA

TBEV-specific IgG antibodies were analysed by using purified formalin-inactivated TBEV strain Neudörfl as described previously [38]. A standard polyclonal human anti-TBE virus serum set at 1000 AU was used for quantification of samples in arbitrary units. Plasma samples were tested twice.

### Neutralization assay

Neutralization assays were carried out in baby hamster kidney cells (ATCC BHK-21) as described previously [39]. Twofold serial dilutions of plasma samples were mixed with 25 TCID50 virus (strain Neudoerfl) and incubated for 1 h at 37°C. BHK-21 cells were then added, and incubation was continued for 3 days. The presence of virus in the supernatant was determined by ELISA. The virus neutralization titre (mean of duplicates) was defined as the reciprocal of the plasma sample dilution that gave a 90% reduction in the absorbance readout in the assay compared to the control without antibody. NT titres ≥10 were considered positive and NT titres <10 negative.

### Statistical analysis

Comparisons of the median fluorescence intensities (MFI) of the fluorescent signal associated with each cytokine in groups of vaccinated and infected subjects were performed using Mann-Whitney test. In addition, group differences were tested by Kolmogorov-Smirnov omnibus tests which gave the same results (IFN-γ, p<0.05; IL-2, p<0.05; TNF-α, p>0.1). The MFI values produced by the different Th1 subtypes were evaluated after log transformation by ANOVA and linear contrasts of all subtypes each against triple positives. Univariate regression analysis was performed to estimate the relationship between TBEV-specific CD4^+^ T cell populations and TBEV-specific neutralizing antibody titers and TBEV-IgG. Comparison of cytokine subset responses between groups of vaccinated and infected subjects was performed by a two-factor ANOVA after arcsine transformation of percentages and removal of the pattern with single TNF-α not considered positive in Boolean analysis. The resulting six groups of lymphocyte patterns were the within-subject factor and the two groups of infected and booster vaccinated subjects were the between-subject factor. All patterns of cytokine expressions were compared within infected and vaccinated groups against the triple positive group by linear contrasts. A chi-square test was used to perform comparisons of Th1 responses to E peptide minipools.

## Results

### Detection of different CD4 cytokine patterns after TBEV infection or vaccination

**Fig 1:**
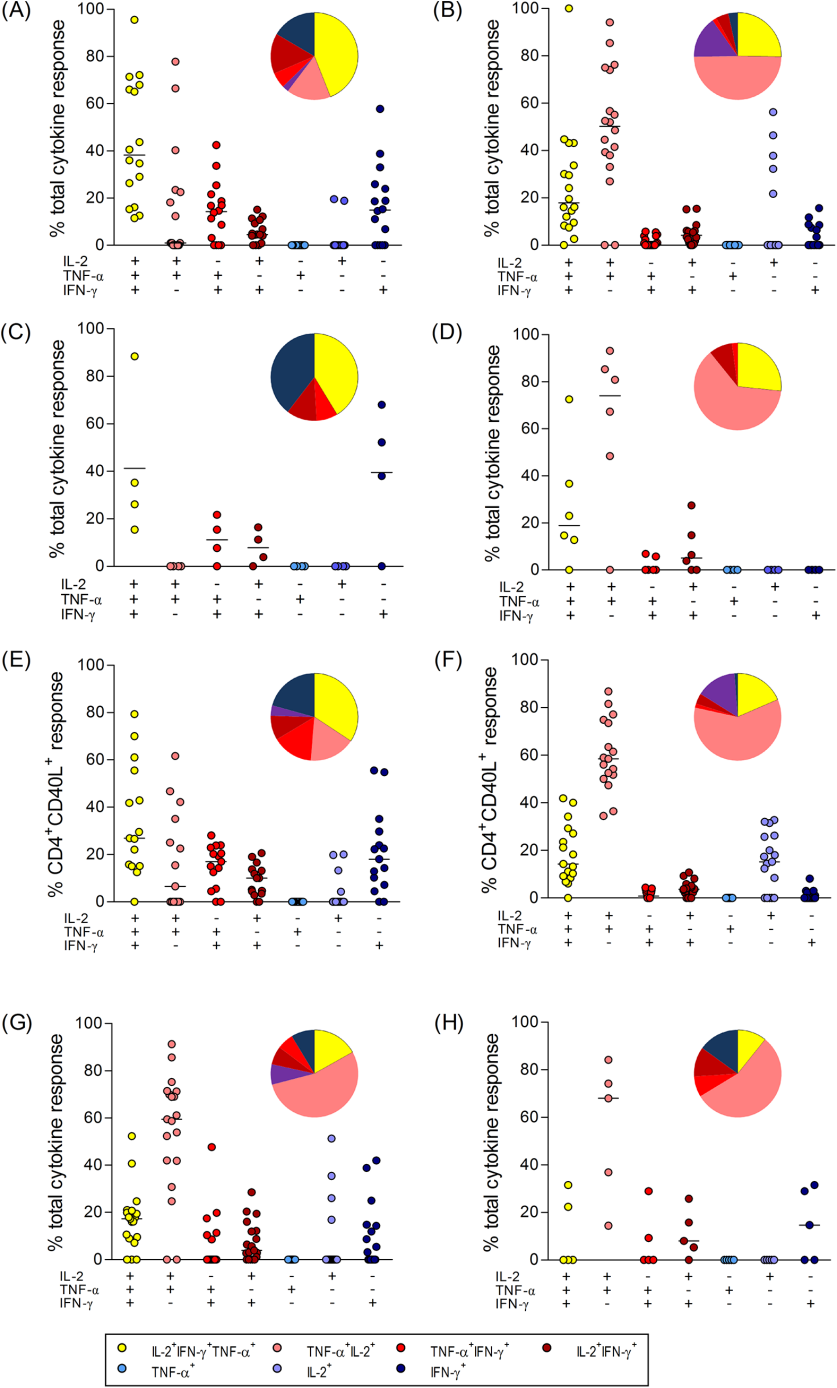
Diverse Th1 cytokine patterns of TBEV-specific CD4^+^ T cells are induced after vaccination or infection. Th1 subtypes, including triple (TNF-α^+^IFN-γ^+^IL-2^+^), dual (TNF-α^+^IFN-γ^+^; TNF-α^+^IL-2^+^; IFN-γ^+^IL-2^+^) and single (TNF-α^+^; IFN-γ^+^; IL-2^+^) cytokine positive CD4^+^ T cells after 6h stimulation of PBMC with E peptide pools in TBE patients (A), TBE booster-vaccinated subjects (B). C peptide pool responses in TBE patients (C) and TBE booster-vaccinated subjects (D). Th1 cytokine patterns after gating on CD40L in TBE patients (E) and booster vaccinated persons (F). Th1 cytokine patterns in TBE primary vaccinated individuals after restimulation with E peptide pools (G) or C peptide pools (H). Medians are indicated by black lines. Pie charts show means of cytokine subsets as a fraction of the total number of positive events in all individuals analysed (pie charts of responses from representative patients, S1 Fig).

For determining TBEV-specific CD4^+^ T cell responses, we analysed the Th1 cytokine expression patterns in PBMC stimulated with peptide pools spanning the entire sequences of the TBEV structural proteins E, prM/M and C. The fractions of TBEV-specific CD4^+^ T cells producing TNF-α, IFN-γ and IL-2 were quantified using Boolean analysis of flow cytometry data that categorized cytokine-positive cells into seven different subsets, including polyfunctional cells (defined as cells producing ≥ 2 cytokines) and single cytokine producing cells (S1 Fig). Fig 1 illustrates the response to E and C peptide pools from TBE patients and TBE vaccinated subjects. PrM/M peptides did not yield a measureable response and cytokine profiles were therefore not included in Fig 1. The pie charts summarize the contribution of cytokine subsets as a fraction of the total number of positive events in all individuals analysed. In total, 16 of 18 TBE patients and 18 of 20 booster-vaccinated subjects showed detectable responses to E (Fig 1A and 1B). Despite substantial variability between individuals in the frequencies of each cytokine combination, a group-wise comparison of the results showed that characteristic cytokine subsets were represented at significantly higher frequencies in a group. The specific response during TBEV infection was dominated by triple-positive IL-2^+^TNF-α^+^IFN-γ^+^ cells (p<0.01; two-factor ANOVA) (Fig 1A), whereas in TBE vaccinees, TNF-α^+^IL-2^+^IFN-γ^-^ and triple positive IL-2^+^TNF-α^+^IFN-γ^+^ cells constituted the largest proportions (p<0.01; two-factor ANOVA) with a trend of higher frequencies of TNF-α^+^IL-2^+^IFN-γ^-^ as compared to triple positive IL-2^+^TNF-α^+^IFN-γ^+^ subsets (p = 0.082; two-factor ANOVA) (Fig 1B). A similar cytokine pattern was seen for C peptide pool responses that could be detected in 4 TBE patients (Fig 1C) and in 6 vaccinated subjects (Fig 1D). Similar polyfunctional cytokine patterns were also obtained when we analysed the subset responses to E peptides after gating CD4^+^ T cells on CD40L which is a highly sensitive marker of antigen-activated CD4^+^ T cells [36, 40] (Fig 1E and 1F).

Since the data obtained from booster-vaccinated subjects represent memory cell responses to multiple TBE vaccinations, we tested whether these were different from responses induced after primary vaccination (2 doses given 1 month apart). CD4^+^ T cells after primary vaccination were therefore analysed for E and C peptide pool responses. As illustrated in Fig 1G and 1H, the composition of Th1 subtypes was very similar to that found in anamnestic responses, indicating that the patterns did not change after booster vaccination.

**Fig 2:**
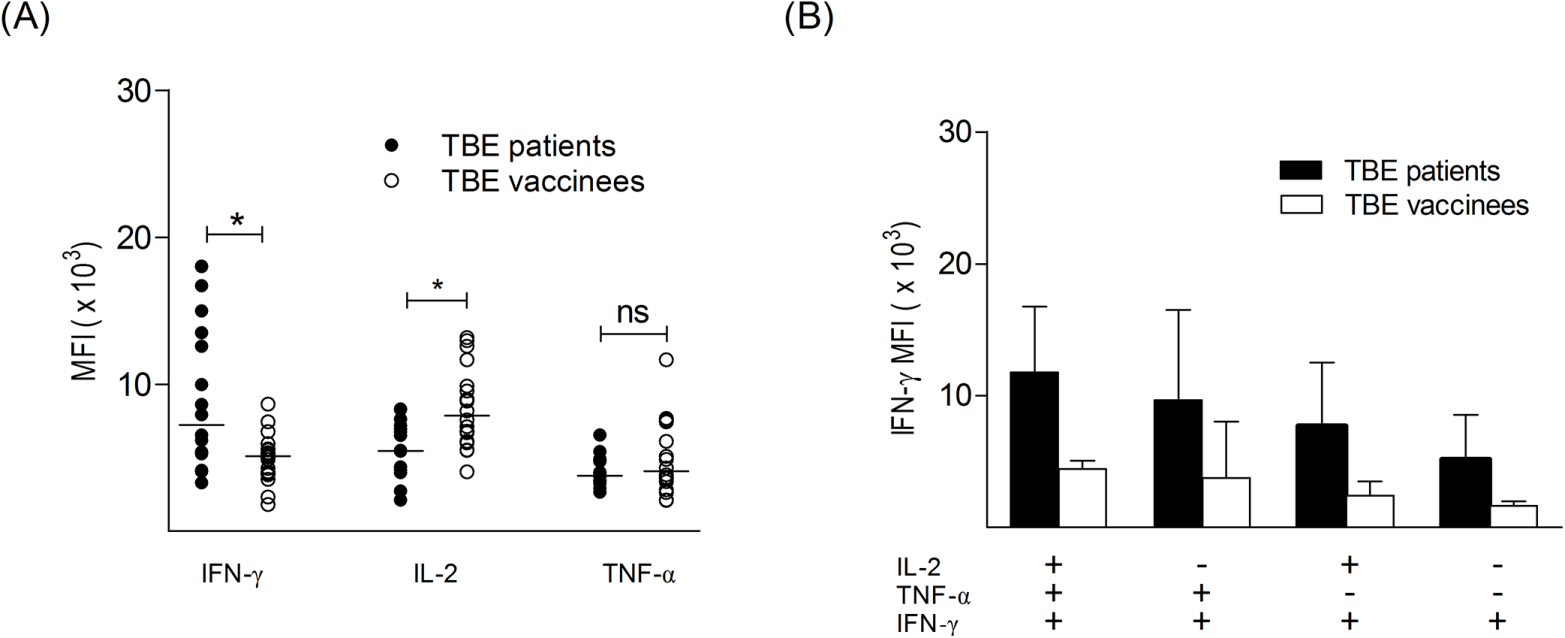
TBEV-specific polyfunctional CD4^+^ T cells from TBE patients and booster vaccinated persons express different amounts of IFN-γ. (A) Median fluorescence intensity (MFI) of IFN-γ, IL-2 and TNF-α in triple-positive TBEV-specific CD4^+^ T cells from TBE patients (n = 16) and booster vaccinated persons (n = 18). Medians are indicated by black lines. Mann Whitney test was used to test for differences between patients and vaccinated groups. (B) MFI (mean + standard deviation) of IFN-γ in IFN-γ^+^ subtypes (TNF-α^+^IFN-γ^+^IL-2^+^; TNF-α^+^IFN-γ^+^; IFN-γ^+^IL-2^+^ and single IFN-γ^+^) from subjects who mounted detectable responses with all 4 IFN-γ subtypes (8 TBE patients and 4 booster-vaccinated subjects). (for individual data, S4 Table).

Previous studies have described a relationship between the frequency of IL-2-producing CD4^+^ T cells and antibody titers with vaccination [17, 18, 41]. Therefore, we analysed the relationship between TBEV-specific CD4^+^ T cell frequencies expressing IL-2, TNF-α or IFN-γ and TBEV-specific IgG and neutralizing antibody titers. Consistent with previous reports, we found a strong correlation of neutralizing antibody titers with IL-2 and TNF-α subsets in TBE-vaccinated persons. In contrast, no significant correlation between these parameters was found in TBE patients (S3 Table). To address the question whether the amount of cytokines produced differed between responder cells from patients and vaccinees, we determined the median fluorescence intensities (MFI) of the fluorescent signal associated with each cytokine in triple-positive cells after restimulation with TBEV E peptide pools. As illustrated in Fig 2A, triple-positive cells from TBE patients displayed significantly higher levels of IFN-γ (p = 0.008, Mann-Whitney U test) and lower IL-2 expression (p = 0.003, Mann-Whitney U test) than those from vaccinated subjects. We also compared the two groups with respect to the amount of IFN-γ produced by the different Th1 subtypes (Fig 2B). In this analysis we included only individuals who mounted detectable responses with all IFN-γ^+^ subtypes (8 TBE patients and 4 booster-vaccinated subjects, S4 Table). In TBE patients, the polyfunctional triple-positive cells produced higher amounts of IFN-γ than single IFN-γ-secreting cells (p = 0.002, ANOVA) and similar differences were found in vaccinees, although at lower levels of IFN-γ production (p = 0.01, ANOVA). In addition, there were no statistical significant differences in the MFI for IL-2 between triple-positive, dual and single IL-2 producing cells from TBE vaccinees (S5 Table).

**Fig 3:**
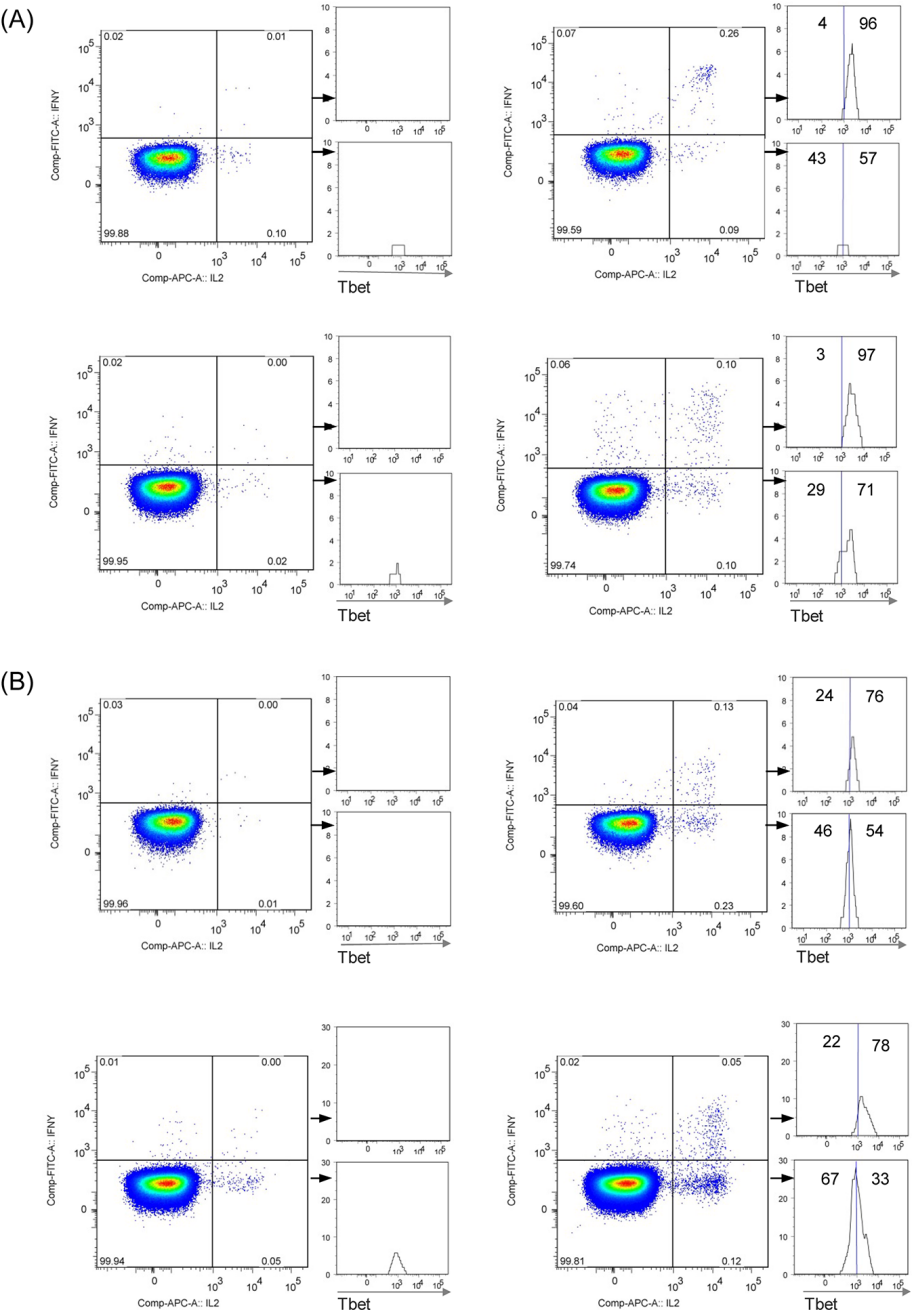
Reduced Th1 lineage transcription factor Tbet expression in TBEV-specific IL-2^+^IFN-γ^-^CD4^+^ T cells. Human PBMC from 2 TBE patients (A) and 2 TBE vaccinated persons (B) were stimulated with TBEV E peptide pools (right panel) or no antigen (left panel). Plots were gated on lymphocytes, live cells, singlets, CD3^+^CD4^+^, CD4^+^CD45RO^+^. Histograms show expression of Tbet in IL-2^+^IFN-γ^-^ (lower quadrants) or IL-2^+^IFN-γ^+^ populations (upper quadrants).

The reduced IFN-γ expression patterns observed in TBE vaccine responses could be due to the presence of Th1 progenitor cells which produce IL-2, but no or low IFN-γ and express lower levels of the transcription factor Tbet [21, 31, 34, 42]. We therefore analysed CD4^+^ T cell responses with respect to Tbet level expression in TBEV-specific IL-2^+^IFN-γ^-^ and IL-2^+^IFN-γ^+^ cells in 2 TBE patients (Fig 3A) and 2 vaccinated subjects (Fig 3B). Individuals of both groups showed high Tbet expression in IL-2^+^IFN-γ^+^ cells, whereas IL-2^+^IFN-γ^-^ cells, predominant in vaccinees, but not in infected subjects, expressed lower Tbet. These data indicate that TBE vaccination generates polyfunctional CD4^+^ T cell populations that differ in their capacity to produce IFN-γ and have distinct Th1 differentiation phenotypes.

### Th cytokine responses to TBEV E-peptide minipools

**Fig 4:**
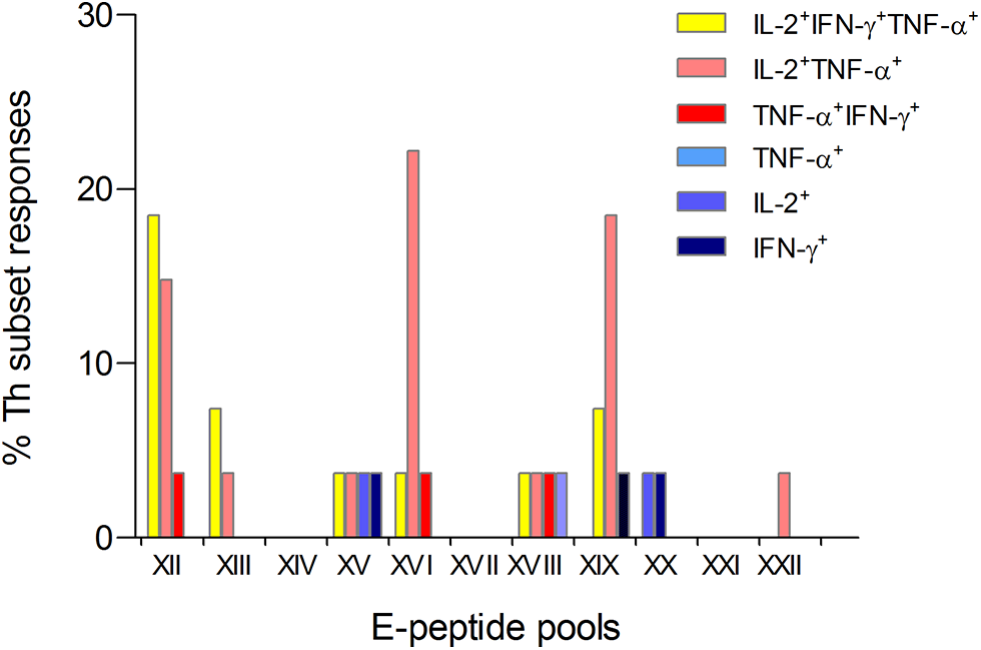
Th1 subtype responses to TBEV E peptide minipools. Percentage of Th subset responses out of all minipool responses from 11 TBE vaccinated subjects (for individual responses, S6 Table).

Our findings that TBE vaccination induced comparatively low IFN-γ responses suggests that studies to determine immunodominant epitopes would not provide sufficient information on the full breadth of the response if they were based on IFN-γ measurements only. This was corroborated by an analysis in which we tested a panel of 122 overlapping peptides, arranged in 11 minipools, each consisting of 11–12 single peptides, spanning the entire sequence of the E protein in PBMC samples from 11 vaccinees (S2 Fig). For TBE patients, sufficient amount of blood samples was not available; therefore, no minipool analysis was performed. As can be seen in Table 1, of the 27 E minipool responses detected with at least one Th subset, 70% were detected with IL-2^+^TNF-α^+^ cells, but only 44% with IFN-γ^+^IL-2^+^TNF-α^+^-cells and ≤ 11% with other Th subtypes (p<0.0001, Chi-square). Overall, IL-2-positive subsets (i.e.: IFN-γ^+^IL-2^+^TNF-α^+^, IFN-γ^+^IL-2^+^, IL-2^+^TNF-α^+^ and IL-2^+^) detected 96% (26/27) of E peptide minipools, whereas IFN-γ^+^subsets (IFN-γ^+^IL-2^+^TNF-α^+^, IFN-γ^+^IL-2^+^, IFN-γ^+^TNF-α^+^, IFN-γ^+^) detected only 48% (13/27) (Table 1). These results demonstrate that more than half of the vaccine responses were IFN-γ-negative and would have been missed in a single IFN-γ assay. For displaying the specific data obtained with the individual minipools, we calculated the frequency of a positive Th subset result for each minipool using the data from all 11 vaccinees obtained from minipool testing. This frequency is displayed as the percentage of Th subset responses out of all minipool responses (Fig 4). Minipools XII, XVI, and XIX most frequently yielded positive results, accounting for 22% (XII, XIX) and 19% (XVI) of the responses in vaccinees. These minipools contain peptides that were previously identified as immunodominant in ELISPOT assays [18]. Minipool XII showed high responder rates for IL-2^+^TNF-α^+^ cells and triple-positive IFN-γ^+^IL-2^+^TNF-α^+^ cells, whereas detection rates of minipools XVI and XIX were 2.5 to 6-fold higher for IL-2^+^TNF-α^+^ cells than for triple-positive IFN-γ^+^IL-2^+^TNF-α^+^ cells. These results confirm that, in vaccinated persons, immunodominant epitopes would be missed using single IFN-γ measurements.

**Table 1:** Analysis of Th1 subtype responses to E peptide minipools in 11 TBE-vaccinated subjects.

Cytokine secreting subtype	Total number of positive E peptide minipool responses	% positive subtype responses
**IFN-γ** ^**+**^ **IL-2** ^**+**^ **TNF-α** ^**+**^	12/27	44%
**IL-2** ^**+**^ **TNF-α** ^**+**^	19/27	70%
**IFN-γ** ^**+**^ **TNF-α** ^**+**^	3/27	11%
**IFN-γ** ^**+**^ **IL-2** ^**+**^	0/27	0%
**TNF-α** ^**+**^	0/27	0%
**IFN-γ** ^**+**^	3/27	11%
**IL-2** ^**+**^	3/27	11%
**IL-2** ^**+**^ **subsets** *	26/27	96%
**IFN-γ** ^**+**^ **subsets** ^#^	13/27	48%

*IL-2-positive subsets (IFN-γ^+^ IL-2^+^TNF-α^+^, IL-2^+^TNF-α^+^, IFN-γ^+^ IL-2^+^, IL-2^+^)

^#^ IFN-γ-positive subsets (IFN-γ^+^ IL-2^+^TNF-α^+^, IFN-γ^+^ IL-2, IFN-γ^+^ TNF-α^+^, IFN-γ^+^)

## Discussion

This study investigated the characteristics of virus-specific CD4^+^ T cells after vaccination with a formalin-inactivated TBE vaccine in comparison to the response raised during natural infection. The data demonstrate that patients with TBEV infection had robust Th1 responses, characterized by polyfunctional cells producing IL-2, TNF-α and IFN-γ. It has been suggested that such polyfunctional cells are likely to be crucial for combating acute virus infection by producing high amounts of IFN-γ, thus contributing to immediate effector function [22, 35, 43]. In vaccinated individuals, however, IFN-γ^+^CD4^+^ T cells constituted a minor population, and these cells expressed significantly lower amounts of IFN-γ per cell compared to those in infected subjects. In contrast to the response observed after infection, the majority of vaccine-induced CD4^+^ T cells had a phenotype of IL-2^+^TNF-α^+^IFN-γ^-^ cells similar to responses observed with other non-live vaccines, such as hepatitis B or tetanus [31, 34, 35, 37, 42]. Although the determinants leading to polyfunctional cytokine expression in human virus-specific CD4^+^ T cells are unclear, the different IFN-γ expression patterns of virus-specific CD4^+^ T cells may reflect an influence of the amount and duration of antigen stimulation and/or extra inflammatory signals provided during viral infection that stimulate innate immune receptors, like TLRs which strongly promote acquisition of Th1 effector function [44, 45]. The requirement of such strong signals to establish polyfunctional Th1 responses is also consistent with studies demonstrating that the live attenuated YF-17D vaccine induces a robust population of multicytokine-producing CD4^+^ T cells [46–48]. The absence of extra inflammatory signals during immunization with non-replicating virus vaccines may provide a selective advantage for the expansion of a large population of IL-2^+^IFN-γ^-^ T cells that proliferate through autocrine IL-2 signaling and could lead to an increased size of the memory cell pool [49]. The higher response magnitudes in TBE vaccinated persons *vs* infected patients observed in a previous study using *ex vivo* IL-2 ELISPOT assays [18] could also be explained by this scenario. Furthermore, we showed that vaccine-induced CD4^+^ T cell populations exhibited different Th1 lineage transcription factor Tbet expression, suggesting that vaccination generates Th1 cell populations with distinct differentiation phenotypes that include both, Th1 effector cells (Tbet^hi^IFN-γ^+^) and Th1 precursors (Tbet^lo^) which function as a pool of memory cells capable of differentiating into Th1 effectors upon subsequent antigen challenge [50]. The findings of distinct Th1 cell populations were obtained with small sample numbers and larger studies are clearly required to confirm the observations made here. Potentially, the phenotype of vaccine-generated responses could change following multiple booster vaccinations. We therefore analysed a data set derived from primary TBE vaccinated subjects to show that this was not the case, because a similarly high proportion of IL-2^+^IFN-γ^-^ cells was induced after primary and anamnestic responses. This finding is in agreement with studies demonstrating that after multiple booster vaccinations with protein subunit vaccines, such as hepatitis B or tetanus, the dominant CD4^+^ T cell responses consisted of cells producing IL-2 and not IFN-γ [34, 37, 42, 51]. The different IFN-γ expression patterns of virus-specific CD4^+^ T cells in vaccinated and infected groups were also observed in response to TBEV C protein, but response magnitudes were lower than those of E-specific responses. However, we previously showed that the extents of reactivities to peptides from E, prM/M and C were concordant with the sizes of these proteins as well as their amounts present in the virion, suggesting a similar propensity for all three proteins to induce a CD4^+^ T cell response [18]. Using TBEV E peptide minipools, we showed that IFN-γ^+^ cells contributed in less than half of the responses in vaccinees. These data indicate that the use of IFN-γ to characterize such responses may strongly underestimate the response magnitude and breadth, since a significant number of epitopes would remain undetected. The findings corroborate other reports indicating that IFN-γ is not sufficient to determine the extent of antigen-specific T cell responses [37, 52, 53].

While there is still little understanding on the role of Th1 lineage subtypes *in vivo*, it is notable that the extent to which different subtypes contributed to the overall response varied considerably between individuals. An analysis of serum TBEV-specific antibody responses showed that, in agreement with previous studies [17, 18], neutralizing antibody titers were strongly correlated with the magnitude of IL-2 and TNF-α responses in vaccinees, suggesting that the expansion of these Th populations was an important component of the immune response to the TBE vaccine. Further studies will be necessary to find out to which extent the individual variation of these Th subtypes contributes to the well-documented variation in the persistence of antibody responses after TBE vaccination [54]. Interestingly, no correlation between cytokine subsets and TBEV-specific antibody titers was found in TBEV patients. The reason for this discrepancy is unclear. Therefore, further studies that address the role of other Th cell subsets in the development of a protective anti-TBE response are needed.

In summary, we show that both TBE vaccination and infection generate polyfunctional Th1 responses, but the cytokine patterns are diverse and characterized by significantly lower IFN-γ^+^ subsets in vaccine responses. As a consequence, only 48% of TBEV peptides were detected in an epitope mapping approach with IFN-γ^+^ cells compared to 96% with IL-2^+^ cells. The results have direct implications for approaches that address epitope specificity and breadth of these responses and provide important insights into the functional mechanisms that coordinate vaccine responses.

## Supporting Information

S1 Fig Flow cytometry analysis of TBEV-specific CD4^+^ T cell responses. CD4^+^ T cells were analyzed for expression of TNF-α, IFN-γ and IL-2 after restimulation with peptide pools covering the entire sequences of the TBEV structural proteins, E, prM/M and C. (A) Gating strategy to identify viable CD3^+^CD4^+^ T cells producing TNF-α, IFN-γ or IL-2. Boolean gates, applied to the three cytokine gates discriminate 7 subtypes with distinct cytokine combinations from one representative TBE-vaccinated person. (B) Responses to TBEV E and C peptide pools in representative samples, each from two TBE patients and two vaccinated persons. Pie charts with slices representing frequencies of the cytokine subsets of total cytokine-positive cells. The arcs represent the proportion of cytokine combinations that express IL-2 (grey) or IFN-γ (blue).
(TIF)

S2 Fig Summary of TBEV E peptides included in minipools. The 122 peptides covered the entire amino acid (aa) sequence of the E protein from TBE virus Neudörfl strain (NCBI GI 27596778). For minipool analysis 11–12 peptides were arranged into minipools XII-XXII.
(TIF)

S1 Table Patient demographics. (DOCX)

S2 Table Fluorescent antibody conjugates. (DOCX)

S3 Table Univariate regression analysis to estimate the relationship between TBEV-specific CD4^+^ T cell populations and TBEV-specific neutralizing antibody titers and TBEV-IgG. (DOCX)

S4 Table Median fluorescence intensity (MFI) of IFN-γ in TBEV-specific CD4^+^ T cells from TBE patients and booster-vaccinated subjects. (DOCX)

S5 Table Median fluorescence intensity (MFI) of IL-2 in TBEV-specific CD4^+^ T cells from TBE booster-vaccinated subjects. (DOCX)

S6 Table TBEV E peptide minipools that induce Th1 subtype responses in TBE vaccinated subjects. (DOCX)
