# Supplementary material for: Human CD4+ T Helper Cell Responses after Tick-Borne Encephalitis Vaccination and Infection
Source: PLoS One. 2015 Oct 14;10(10):e0140545. doi: 10.1371/journal.pone.0140545 (PMC4605778; doi:10.1371/journal.pone.0140545)
Supplement: S1 Table — (DOCX) [file pone.0140545.s003.docx]

**S1 Table. Patient demographics.**

| **Subject #** | **age** | **sex** | **TBEV infection** | **TBE vaccination** | **Date of last TBE booster vaccination** | **Date of blood sampling** |
| --- | --- | --- | --- | --- | --- | --- |
| 104 | 43 | male | 2009 | no | - | 03.06.2009 |
| 109 | 30 | female | 2009 | no | - | 23.06.2009 |
| 112 | 57 | male | 2009 | no | - | 07.07.2009 |
| 113 | 81 | male | 2009 | no | - | 03.07.2009 |
| 120 | 57 | male | 2009 | no | - | 23.07.2009 |
| 121 | 52 | male | 2009 | no | - | 28.07.2009 |
| 122 | 23 | male | 2009 | no | - | 28.07.2009 |
| 123 | 25 | female | 2009 | no | - | 28.07.2009 |
| 124 | 59 | male | 2009 | no | - | 31.07.2009 |
| 125 | 25 | female | 2009 | no | - | 06.08.2009 |
| 129 | 55 | male | 2009 | no | - | 24.09.2009 |
| 134 | 54 | male | 2009 | no | - | 24.11.2009 |
| 101 | 67 | male | 2009 | no | - | 15.05.2009 |
| 201 | 60 | female | 2008 | no | - | 27.08.2008 |
| 202 | 44 | male | 2008 | no | - | 19.12.2008 |
| 119 | 59 | female | 2009 | no | - | 27.04.2010 |
| 203 | 56 | female | 1987 | no | - | 23.12.2008 |
| 204 | 52 | female | 1986 | no | - | 03.03.2009 |
| 3 | 62 | female | no | yes | 15.04.2009 | 21.04.2009 |
| 5 | 74 | male | no | yes | 21.04.2009 | 06.05.2009 |
| 6 | 64 | female | no | yes | 21.04.2009 | 06.05.2009 |
| 11 | 63 | female | no | yes | 20.04.2009 | 04.05.2009 |
| 14 | 70 | female | no | yes | 27.04.2009 | 07.05.2009 |
| 16 | 60 | female | no | yes | 23.04.2009 | 06.05.2009 |
| 17 | 24 | male | no | yes | 03.03.2011 | 21.03.2011 |
| 23 | 62 | male | no | yes | 06.05.2009 | 13.05.2009 |
| 27 | 62 | female | no | yes | 12.05.2009 | 19.05.2009 |
| 31 | 60 | female | no | yes | 09.06.2009 | 18.06.2009 |
| 34 | 67 | female | no | yes | 15.06.2009 | 22.06.2009 |
| 51 | 50 | male | no | yes | 18.04.2011 | 02.05.2011 |
| 52 | 46 | male | no | yes | 18.04.2011 | 02.05.2011 |
| 53 | 21 | female | no | yes | 12.05.2011 | 26.05.2011 |
| 54 | 26 | female | no | yes | 02.12.2009 | 07.01.2010 |
| 55 | 24 | male | no | yes | 12.04.2011 | 29.04.2011 |
| 56 | 54 | male | no | yes | 07.05.2009 | 15.05.2009 |
| 57 | 24 | female | no | yes | 05.10.2009 | 25.11.2009 |
| 58 | 25 | female | no | yes | 17.05.2011 | 31.05.2011 |
| 59 | 44 | female | no | yes | 18.04.2011 | 02.05.2011 |
