# Supplementary material for: Human CD4+ T Helper Cell Responses after Tick-Borne Encephalitis Vaccination and Infection
Source: PLoS One. 2015 Oct 14;10(10):e0140545. doi: 10.1371/journal.pone.0140545 (PMC4605778; doi:10.1371/journal.pone.0140545)
Supplement: S2 Table — (DOCX) [file pone.0140545.s004.docx]

**S2 Table. Fluorescent antibody conjugates**

| **Antigen** | **Fluorochrome** | **Clone** | **Supplier** |
| --- | --- | --- | --- |
| CD3 | APC-H7 | SK7 | BD |
| CD4 | Pacific Blue | RPA-T4 | BD |
| Live/Dead | Aqua-fluorescent reactive dye | - | Invitrogen |
| IL-2 | APC | 5344.111 | BD |
| TNF-α | PE-Cy7 | Mab11 | BD |
| IFN-γ | FITC | 25723.11 | BD |
| CD154 (CD40L) | PE | TRAP1 | BD |
| CD45RO | PE-Cy7 | UCHL1 | BD |
| Tbet | PE | 4B10 | eBioscience |
