# Supplementary material for: Human CD4+ T Helper Cell Responses after Tick-Borne Encephalitis Vaccination and Infection
Source: PLoS One. 2015 Oct 14;10(10):e0140545. doi: 10.1371/journal.pone.0140545 (PMC4605778; doi:10.1371/journal.pone.0140545)
Supplement: S3 Table — (DOCX) [file pone.0140545.s005.docx]

**S3 Table. Univariate regression analysis to estimate the relationship between TBEV-specific CD4^+^ T cell populations and TBEV-specific neutralizing antibody titers and TBEV-IgG**.

| **Subject group** | **Predictor** | **TBEV-neutralizing antibody** | | **TBEV-IgG (ELISA)** | |
| --- | --- | --- | --- | --- | --- |
|  |  | **Coefficient** | ***p* value** | **Coefficient** | ***p* value** |
| **Booster vaccinated subjects** | IL-2 | 0.773 | 0.005 | 0.576 | 0.064 |
|  | TNF-α | 0.756 | 0.007 | 0.589 | 0.056 |
|  | IFN-γ | 0.608 | 0.047 | 0.469 | 0.146 |
| **TBE patients** | IL-2 | 0.349 | 0.242 | 0.251 | 0.390 |
|  | TNF-α | 0.431 | 0.148 | 0.329 | 0.261 |
|  | IFN-γ | 0.562 | 0.070 | 0.458 | 0.135 |

Standardized coefficients and p-value of log TBEV-specific CD4^+^ T cells expressing IL-2, TNF-α and IFN-γ on log TBEV- neutralizing antibody titers and log TBEV-IgG (VIE U/ml) in TBE booster vaccinated persons or TBE patients.
