# Supplementary material for: Human CD4+ T Helper Cell Responses after Tick-Borne Encephalitis Vaccination and Infection
Source: PLoS One. 2015 Oct 14;10(10):e0140545. doi: 10.1371/journal.pone.0140545 (PMC4605778; doi:10.1371/journal.pone.0140545)
Supplement: S4 Table — (DOCX) [file pone.0140545.s006.docx]

**S4 Table. Median fluorescence intensity (MFI) of IFN-γ in TBEV-specific CD4^+^ T cells in TBE patients and booster-vaccinated subjects**

| **Subject group** | **Subject #** | **Median fluorescence intensity (MFI) IFN-γ** | | | |
| --- | --- | --- | --- | --- | --- |
|  |  | **IFN-γ^+^TNF-α^+^IL-2^+^** | **IFN-γ^+^TNF-α^+^** | **IFN-γ^+^IL-2^+^** | **IFN-γ^+^** |
| TBE patients | 109 | 6179 | 3443 | 5017 | 2508 |
|  | 123 | 14983 | 4417 | 2519 | 2079 |
|  | 124 | 13498 | 16531 | 12013 | 11025 |
|  | 125 | 16695 | 16943 | 16229 | 3543 |
|  | 134 | 6537 | 6438 | 3256 | 1959 |
|  | 101 | 5396 | 7947 | 4257 | 8409 |
|  | 201 | 18017 | 19367 | 10370 | 7430 |
|  | 202 | 12578 | 6058 | 7649 | 4195 |
| Booster vaccinated subjects | 3 | 4046 | 8269 | 2123 | 2104 |
|  | 16 | 4882 | 6537 | 4009 | 1593 |
|  | 54 | 3832 | 126 | 1700 | 1515 |
|  | 57 | 3925 | 29 | 1735 | 1268 |
