# Supplementary material for: Human CD4+ T Helper Cell Responses after Tick-Borne Encephalitis Vaccination and Infection
Source: PLoS One. 2015 Oct 14;10(10):e0140545. doi: 10.1371/journal.pone.0140545 (PMC4605778; doi:10.1371/journal.pone.0140545)
Supplement: S5 Table — (DOCX) [file pone.0140545.s007.docx]

**S5 Table. Median fluorescence intensity (MFI) of IL-2 in TBEV-specific CD4^+^ T cells from TBE booster-vaccinated subjects**

| **Subject group** | **Subject #*** | **Median fluorescence intensity (MFI) IL-2** | | | |
| --- | --- | --- | --- | --- | --- |
|  |  | **IFN-γ^+^TNF-α^+^IL-2^+^** | **IL-2^+^TNF-α^+^** | **IL-2^+^IFN-γ^+^** | **IL-2^+^** |
| Booster vaccinated subjects | 3 | 13171 | 13211 | 10119 | 9814 |
|  | 5 | 6698 | 6547 | 1058 | 1857 |
|  | 14 | 11650 | 10991 | 8526 | 7826 |
|  | 31 | 4034 | 4377 | 1384 | 2655 |
|  | 56 | 6781 | 7510 | 2263 | 2525 |

*The analysis included only individuals who mounted detectable responses with all four IL-2-positive subsets (IFN-γ^+^ IL-2^+^TNF-α^+^, IL-2^+^TNF-α^+^, IFN-γ^+^ IL-2^+^, IL-2^+^)
