# Supplementary material for: Human CD4+ T Helper Cell Responses after Tick-Borne Encephalitis Vaccination and Infection
Source: PLoS One. 2015 Oct 14;10(10):e0140545. doi: 10.1371/journal.pone.0140545 (PMC4605778; doi:10.1371/journal.pone.0140545)
Supplement: S6 Table — (DOCX) [file pone.0140545.s008.docx]

**S6 Table.** **TBEV E peptide minipools that induce Th1 subtype responses in TBE vaccinated subjects**

| **Subject group** | **Subject #** | **Th cytokine subsets** | | | | | | | | **Total positive minipool responses** | **Minipool responses of IL-2^+^ subsets*** | **Minipool responses of IFN-γ^+^ subsets^#^** |
| --- | --- | --- | --- | --- | --- | --- | --- | --- | --- | --- | --- | --- |
|  |  | **IL-2^+^TNF-α^+^IFN-γ^+^** | **IL-2^+^TNF-α^+^** | **IL-2^+^IFN-γ^+^** | **TNF-α^+^IFN-γ^+^** | **TNF-α^+^IFN-γ^+^** | **TNF-α^+^** | **IL-2^+^** | **IFN-γ^+^** |  |  |  |
| Booster vaccinated | 3 | - | XII, XV, XVI, XIX, XXII | - | - | - | - | - | - | 5 | 5 | - |
|  | 6 | XIX | XVI, XIX | - | - | - | - | - | XX | 3 | 2 | 2 |
|  | 17 | XII | XII, XVI |  |  |  |  |  |  | 2 | 2 | 1 |
|  | 54 | - | - | - | - | - | - | - | - | - | - | - |
|  | 55 | XII, XIII, XV, XVI, XVIII, XIX | XVI, XVIII, XIX | - | XVI, XVIII | - | - | - | XV | 6 | 6 | 6 |
|  | 58 | - | - | - | - | - | - | - | - | - | - | - |
| Primary vaccinated | 1 | XII | XII, XVI, XIX | - | - | - | - | - | - | 3 | 3 | 1 |
|  | 2 | XII, XIII | XII, XIII, XVI, XIX | - | XII | - | - | - | - | 4 | 4 | 2 |
|  | 3 | - | - | - | - | - | - | XV | - | 1 | 1 | - |
|  | 23 | XII | - | - | - | - | - | XVIII, XX | - | 3 | 3 | 1 |
|  | 25 | - | - | - | - | - | - | - | - | - | - | - |

*IL-2-positive subsets (IFN-γ^+^IL-2^+^TNF-α^+^, IFN-γ^+^IL-2^+^, IL-2^+^TNF-α^+^, IL-2^+^).

^#^IFN-γ-positive subsets (IFN-γ^+^IL-2^+^TNF-α^+^, IFN-γ^+^IL-2^+^, IFN-γ^+^TNF-α^+^, IFN-γ^+^)
